# An efficient computer-aided structural elucidation strategy for mixtures using an iterative dynamic programming algorithm

**DOI:** 10.1186/s13321-017-0244-9

**Published:** 2017-11-15

**Authors:** Bo-Han Su, Meng-Yu Shen, Yeu-Chern Harn, San-Yuan Wang, Alioune Schurz, Chieh Lin, Olivia A. Lin, Yufeng J. Tseng

**Affiliations:** 1 Department of Computer Science and Information Engineering, National Taiwan University, No. 1 Sec. 4, Roosevelt Road, Taipei, 106 Taiwan; 2 Graduate Institute of Networking and Multimedia, National Taiwan University, No. 1 Sec. 4, Roosevelt Road, Taipei, 106 Taiwan; 3 Graduate Institute of Biomedical Electronics and Bioinformatics, National Taiwan University, No. 1 Sec. 4, Roosevelt Road, Taipei, 106 Taiwan

**Keywords:** CASE, Natural products, Dynamic programming, Polynomial time

## Abstract

The identification of chemical structures in natural product mixtures is an important task in drug discovery but is still a challenging problem, as structural elucidation is a time-consuming process and is limited by the available mass spectra of known natural products. Computer-aided structure elucidation (CASE) strategies seek to automatically propose a list of possible chemical structures in mixtures by utilizing chromatographic and spectroscopic methods. However, current CASE tools still cannot automatically solve structures for experienced natural product chemists. Here, we formulated the structural elucidation of natural products in a mixture as a computational problem by extending a list of scaffolds using a weighted side chain list after analyzing a collection of 243,130 natural products and designed an efficient algorithm to precisely identify the chemical structures. The complexity of such a problem is NP-complete. A dynamic programming (DP) algorithm can solve this NP-complete problem in pseudo-polynomial time after converting floating point molecular weights into integers. However, the running time of the DP algorithm degrades exponentially as the precision of the mass spectrometry experiment grows. To ideally solve in polynomial time, we proposed a novel iterative DP algorithm that can quickly recognize the chemical structures of natural products. By utilizing this algorithm to elucidate the structures of four natural products that were experimentally and structurally determined, the algorithm can search the exact solutions, and the time performance was shown to be in polynomial time for average cases. The proposed method improved the speed of the structural elucidation of natural products and helped broaden the spectrum of available compounds that could be applied as new drug candidates. A web service built for structural elucidation studies is freely accessible via the following link (http://csccp.cmdm.tw/).

**Electronic supplementary material:**

The online version of this article (10.1186/s13321-017-0244-9) contains supplementary material, which is available to authorized users.

## Background

Examining natural and therapeutic products is crucial for drug development because many chemically synthesized compounds have potentially serious toxicity and adverse effects, while less toxic compounds extracted from natural products could possibly be developed into new drug candidates [1]. In addition, natural products often open new chemical spaces not explored by synthetic compounds produced by combinatorial chemistry and can further expand the diversity and novelty of molecules by extracting different natural sources, such as the deep and cold seas [2, 3]. A review by Newman and Cragg [2] indicated that 47% of new anti-cancer drugs from 1950 to 2006 were originally from or derived from natural products. Recently, Butler et al. [3] reviewed 100 natural products and natural products-derived compounds that were either evaluated in clinical trials or in registration at the end of 2013. They concluded that 50% of the compounds were natural products or semi-synthetic natural products, while the remaining compounds were classified as natural products-derived compounds. The exploration of new lead compounds from natural products and their successful development into clinical trials will continue to be a significant trend in drug discovery over the next few years.

However, natural products-based drug discovery faces many challenges [4], and the exploration of natural products for new drug development was actually disfavored by the pharmaceutical industry in the 2000s [5]. One of the major hurdles is the extremely time-consuming processes involved in the isolation and structural elucidation of bioactive compounds from natural products composed of complicated mixtures. Because the magnitude of the natural products database is limited, high-throughput screening methods cannot be used to effectively identify potential natural products drugs. Many advances in mass spectrometry (MS) and nuclear magnetic resonance (NMR) automation techniques over the last two decades have accelerated structural elucidation processes for complex natural products mixtures. MS is a common tool used to identify elemental constituents of a molecule. MS data can provide the molecular weight, fragmentation pattern, and molecular formula, which can then be matched to structures. Current advances in MS instruments can provide high-resolution molecular weight measurements [6] and reduce the total number of overlapping m/z values. However, MS data itself is insufficient to determine the structure of a partially or completely unknown compound [7–9]. On the other hand, NMR methods can give a spectroscopic overview of compounds. Although high-resolution and high-dimensional NMR methods have undergone continual advancement [10–12], NMR still cannot independently elucidate novel chemical structures unless co-eluting compounds can be completely separated [8]. Even though LC–NMR–MS [13] and LC–UV–solid-phase extraction–NMR–MS [9] have proven to be effective methods to elucidate compound structures in natural products extracts, the successful structural elucidation of unknown compounds still greatly depends on the development of computational systems to help evaluate the mass spectral data [14].

Computer-aided structure elucidation (CASE), developed 40 years ago [15–18], is a well-known computational approach that can accelerate the process of identifying possible chemical constituents based on expert systems. To fully automate structural elucidation via MS and NMR techniques, different advanced algorithms have been applied in CASE [19–23]. However, the many limitations [24] of CASE expert systems still hinder the creation of fully automatic processes for structural elucidation [25]. One of the major restrictions is the requirement of 2D NMR data as input in these CASE systems [26]. 2D NMR experiments are intrinsically insensitive [27] and extremely time-consuming, especially for the extraction of ^13^C nucleus peak shifts [25]. Moreover, inaccurate structural elucidation may result from the co-elution of compounds that cannot be completely separated. Therefore, current traditional CASE tools based on 2D NMR spectra still cannot meet the structure-solving needs of experienced natural product chemists [4]. Using MS to develop CASE systems can provide more significant benefits than NMR-based CASE systems, as MS is more sensitive, and the amount of unknown structures in the mixtures needed for analysis is smaller. Furthermore, MS is not usually affected by impurities in the input mixture [28].

Harn et al. [29] proposed a novel CASE algorithm, known as *NP*-*StructurePredictor*, to predict individual components in an natural product mixture strictly using information obtained from LC–MS experiments. The purpose of *NP*-*StructurePredictor* is to generate possible chemical structures to aid in the identification or structural elucidation of unknown natural products. This can be achieved by matching a list of known scaffolds with a list of weighted side chains to produce a list of possible compounds under specified structural constraints. In this study, we convert this structural elucidation problem to a formal mathematic formula and refer to the problem as the *Chemical Substituents*-*Core Combinatorial Problem* (*CSCCP*). Since the computational complexity of the *CSCCP* is NP-complete, the search for optimal solutions (valid structures) in the *CSCCP* must be executed in exponential time complexity for any deterministic algorithms without a loss of generality. Thus, using brute force (BF) algorithms, which search all possible answers, to solve the *CSCCP* cannot be finished in a reasonable timeframe. In *NP*-*StructurePredictor*, a branch and bound (BB) strategy was applied to search for and generate a set of optimal solutions. Nevertheless, the BB strategy has its limitations as well: [1] although the execution time is shorter, in many cases, the algorithm still cannot be finished in a reasonable timeframe, [2] it is difficult to analyze the stability and accuracy of the BB method, and [3] the experimental execution time in real cases is unstable and poor for complex mixtures and sometimes even as slow as the BF algorithm. The BB algorithm used in *NP*-*StructurePredictor* still limits the number of combinatorial candidates of the side chains for each scaffold so that the program can be finished in a reasonable execution time. In such cases, *Structure Hunter* cannot find the optimal solutions.

In this study, to further promote the performance of searching for all optimal solutions of the *CSCCP* problem, we first present a pseudo-polynomial time algorithm based on classical dynamic programming (DP) strategies that can effectively and accurately search for all correct structures in a natural product mixture. The DP strategy is based on the method used by Ibarra and Kim [30]. However, because this is a pseudo-polynomial time algorithm, the time performance is limited by the required precision of the molecular weight, which is between four and six decimal places. We then propose another iterative DP algorithm that can be executed in polynomial time for the average case. Four complex herbs with verified structures were applied in the study of *NP*-*StructurePredictor*, and all were successfully predicted by this method. We compared the time performance of our algorithm in *NP*-*StructurePredictor*. For all cases, our iterative DP algorithm outperformed the BF algorithm, classical DP algorithm, and the BB program in *NP*-*StructurePredictor* and also could run to completion in a reasonable time and provide optimal solutions. We developed a new, efficient CASE strategy that can accurately predict the possible structures of compounds in mixtures based only on information obtained from LC–MS experiments.

## Results and discussion

The identification or prediction of the main chemical components present in an natural product mixture with traditional chromatographic methods is time-consuming. The limited compound references also make identification or prediction more difficult for each constituent in a mixture. Hence, an efficient algorithm to solve the *CSCCP* is needed.

This section reports the simulation results of the DP algorithms developed in this paper. Two other traditional methods were implemented and compared with the DP algorithms in terms of quality and time performance. All four of the algorithms were implemented in Java (JDK version 7) and tested on a Linux PC with an Intel Xeon(R) CPU 2.40 GHz with 32 GB of memory. Four types of natural products were used in our simulation: *Cuscuta chinensis* (*C. chinensis*), *Ophiopogon japonicus* (*O. japonicus*), *Polygonum multiflorum* (*P. multiflorum*), and *Angelica* sp. According to the experimental identification procedures from the Natural Product Laboratory of the Taiwan Medical and Pharmaceutical Industry Technology and Development Center as well as investigations from earlier publications, the main structures of each herb have been established and were used as a validation set for the evaluation of our simulation results. The time complexity analysis of our new CASE algorithms, quality of the search results, and running time performance compared to those of the traditional methods for the four cases were also discussed in the last sections.

Targeted MWs and seed scaffolds are necessary input information in our algorithm. To obtain possible targeted MWs, LC–MS experiments for the tested natural products were performed beforehand. Targeted MWs can then be retrieved from peak tables generated in LC–MS experiments. In our cases, we used MAVEN [31] or XCMS [32] to extract peak tables and then retrieved all possible molecular weights as input targeted MWs. To identify the main structures of each tested natural products, the obtained data including parent and daughter ions pattern were compared with the compounds spectra of similar medicinal herbs in earlier publications or databases. This step resulted in the preliminary identification of top five high intensity peaks in our cases. These peaks can be validated with known standard compounds analyzed under the same LC conditions, by comparing and matching the retention times and MS/MS spectra. These identified main structures were used as our validation sets. The remaining core structures can be used directly as the seed scaffolds in our tested cases after all terminal side chains of the identified structures were eliminated. The input targeted MWs and seed scaffolds of the four natural products were listed in the following sections.

The choice of the *seed scaffolds* is likely to influence the outcome of the structural elucidation. In real cases, users can roughly identify the similar structures of natural products, in which compound spectra are similar to all high intensity peaks measured in the preliminary LC–MS experiments. Because those identified structures can be regarded as potential candidates of individual component in test materials, the core structures of these candidates can be directly used as seed scaffolds in our algorithm. Furthermore, when users cannot provide the seed scaffolds of the test material, our algorithm is capable of performing a full search on all our collected 83,242 possible scaffolds. The full searching strategy can automatically generate suitable candidates and run to completion in a reasonable timeframe.

### Structural elucidation of the four natural products using the iterative DP algorithm

**Fig. 1:**
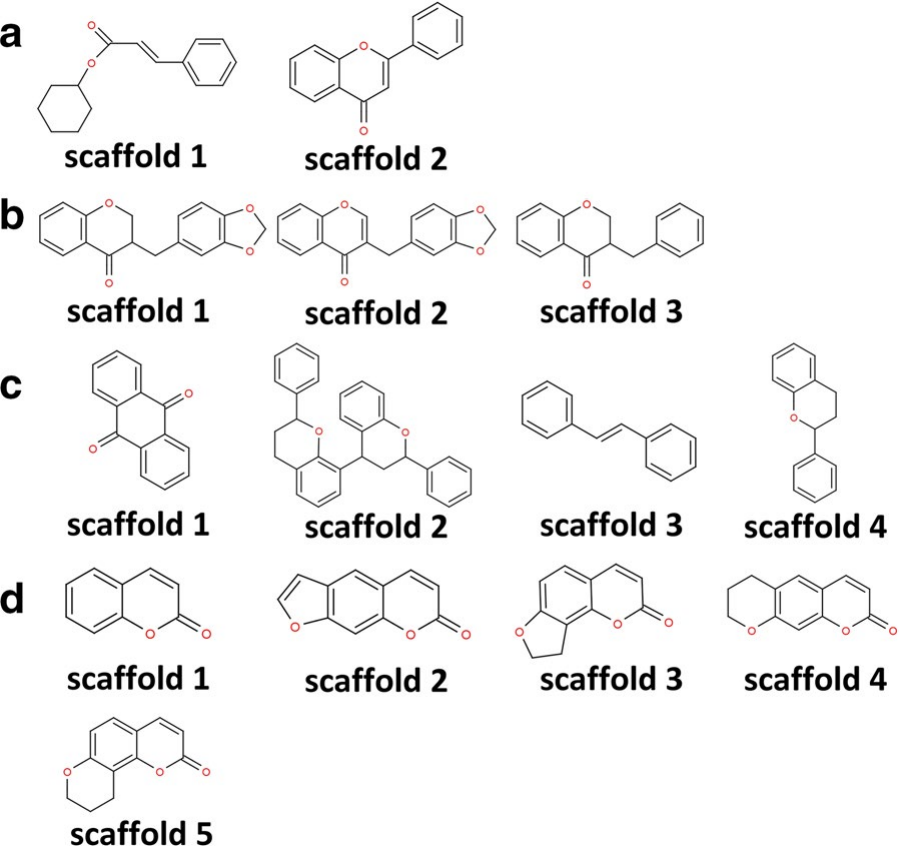
The seed scaffolds used for structural elucidation of the four tested natural products in our DP algorithms: **a** *C. chinensis*, **b** *O. japonicus*, **c** *P. multiflorum*, and **d** *Angelica* sp.

To identify the main structures in an natural product mixture using the iterative DP algorithm, users must first provide the program with a list of target molecular weights (*targeted MWs*) in a tested natural product obtained from the LC–MS spectra as well as *seed scaffolds* if the user has prior knowledge of the potential structural category for compounds in the natural products. In this study, the *targeted MWs* extracted from the mass spectra of the four natural products are listed in Table 1. *C. chinensis* and *O. japonicus* have four *targeted MWs* each, and *P. multiflorum* contains six *targeted MWs*. For *Angelica* sp., 35 *targeted MWs* are given, and thus, the computational time for *Angelica* sp. is much longer than that for the other tested herbs. In Fig. 1, we illustrate the known *seed scaffolds* considered in the four natural products. *Angelica* sp. has the highest number of *seed scaffolds*. In the DP algorithm, we elucidate unknown chemical structures by extending appropriate side chains on the given *seed scaffolds*. When the program analyzes which side chains can be linked to the *seed scaffolds*, we must also consider the possible sets of positions on the scaffold that can be linked by the side chains. Given one *targeted MW* and *seed scaffold*, our algorithm will output a set of optimal structures identified by applying each possible set of positions on the scaffold to the searching procedure of the DP algorithm. Therefore, a *CSCCP* program needs to be executed $N_{c} \times N_{p} \times N_{k}$ times to identify the main structures for an natural product, where *N*_*c*_ is the number of given *seed scaffolds*, *N*_*p*_ denotes the number of given *target MWs*, and $N_{k}$ represents an average number of possible sets of positions on the *seed scaffolds*. From the statistics of the collected database compounds, the average $N_{k}$ is approximately 5. The number of different types of possible sets of positions ($N_{k}$) in the four tested natural products is listed in Table 2. Among the four herbs, *C. chinensis* in scaffold 1 has the maximum $N_{k}$ of 13, and in scaffold 2, *C. chinensis* has the maximum $N_{k}$ of 231. Since four different *targeted MWs* of *C. chinensis* were considered in this study, in total 4 × (231 + 13) = 976 *CSCCPs* must be executed to elucidate the main structures in *C. chinensis*.

**Table 1:** List of the target molecular weights for the four tested natural products

Case name	Target weight lists
*C. chinensis*	286.24, 302.24, 354.31, 478.41
*O. japonicus*	328.32, 342.35, 356.33, 370.36
*P. multiflorum*	270.24, 284.27, 290.27, 406.39, 432.38, 578.53
*Angelica* sp.	162.03, 186.03, 192.04, 202.03, 216.04, 230.09, 244.11, 246.05, 246.09, 246.09, 270.09, 286.08, 288.10, 300.10, 316.09, 328.13, 334.11, 354.15, 360.08, 360.16, 366.22, 374.14, 376.15, 378.17, 386.14, 388.15, 402.13, 414.17, 414.20, 426.17, 426.17, 428.18, 546.26, 574.29

**Table 2:** Number of different types of possible sets of substituted positions (${\mathbf{N}}_{{\mathbf{k}}}$) for each tested natural product

Case name	(Scaffold number, ${\text{N}}_{\text{k}}$)
*C. chinensis*	(1, 13), (2, 231)
*O. japonicus*	(1, 4), (2, 6), (3, 31)
*P. multiflorum*	(1, 68), (2, 28), (3, 68), (4, 81)
*Angelica sp*.	(1, 58), (5, 28), (3, 8), (4, 15), (5, 11)

We took *C. chinensis* with a *targeted MW* of 286.24 as an example to demonstrate the identification of the main structures using the *IDPforCSCCP* algorithm. We executed the *CSCCP* algorithm 231 times using different sets of possible substituted positions on the second scaffold of *C. chinensis* and obtained 2310 recognized structures. The top 10 ranking results among the 2310 structures are presented in Table 3. The first column in Table 3 provides the ranks according to the probabilities ($\prod\nolimits_{i = 1}^{n} {P_{{i,x_{i} }} }$) of potential combinations of the scaffolds and side chains analyzed from our large collections of natural products in descending order. The second column gives the identified main structures in *C. chinensis* with a *targeted MW* of 286.24. The numbers shown below the structures provide the molecular weight and probability, $\prod\nolimits_{i = 1}^{n} {P_{{ix_{i} }} }$, of the identified structure. The highest two identified structures (rank 1 and rank 2 structures) are precisely the same as the main structures validated by experimental methods. The two main structures in *C. chinensis* with a *targeted MW* of 286.24 were correctly predicted with the highest ranks using the *IDPforCSCCP* algorithm. The predicted results that match the validated structures of *C. chinensis* for all considered *targeted MWs* are also given in Table 4. The first two columns in Table 4 provide the molecular weights of the predicted structures and the corresponding *seed scaffolds*. The third column provides the identified structures that are equal to the validated structures, and their ranks are provided in the last column. All validated main structures in *C. chinensis* are correctly predicted to be in the top 10 ranks. The predicted results for *O. japonicus*, *P. multiflorum*, and *Angelica* sp. are also listed in Additional file 1: Tables S1–S3. In Additional file 1: Table S3, the *seed scaffolds* and the predicted structures are omitted, and the second column only provides the *seed scaffold* number as denoted in Fig. 1. In the “rank” column of Additional file 1: Table S3, “Non” indicates that the expected main structures cannot be identified by our program because the correct side chains that link the *seed scaffold*s were not present in our database. Only 9 out of 62 validated main structures in the four tested natural products cannot be automatically identified by our CASE method. Furthermore, the ranks of the predicted structures listed in Table 4 and Additional file 1: Tables S1–S3 confirmed the accuracy of our algorithm. Among the four tested natural products, the predicted ranks of all the validated structures ranged from 1 to 83, and the average value of the predicted ranks is 8. In our DP algorithm, *DPforCSCCP*, we can thus tentatively set *R* equal to 10 because we can elucidate most of the correct main structures in the four tested natural products using our novel DP CASE algorithm, *IDPforCSCCP,* when *R* is set to 10. The four case studies demonstrated that the structural elucidation functionality of *IDPforCSCCP* is applicable and reliable. Moreover, our ranking methodology was reasonable to reduce false positive identifications because our predicted structures that matched with validated compounds all resulted in high rankings.

**Table 3:**
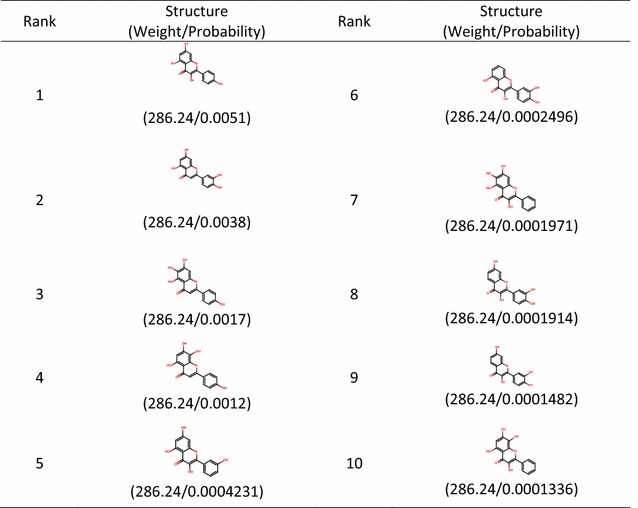
The top ten optimal results from *seed scaffold* 2 with a given weight of 286.24 for *C. chinensis*

**Table 4:**
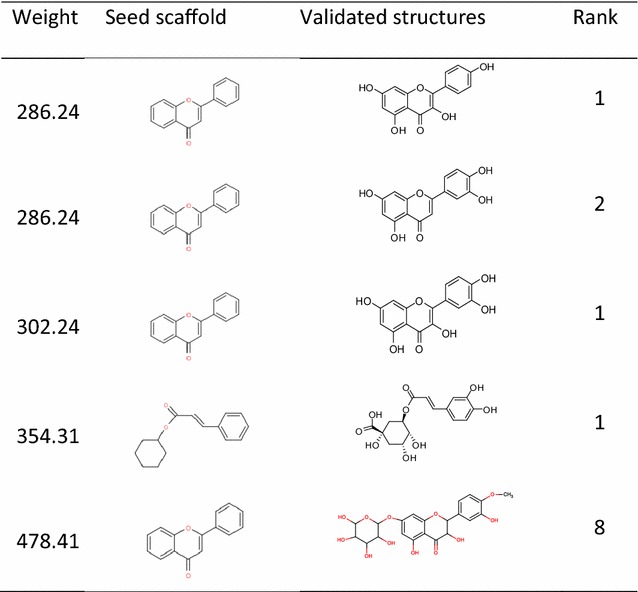
The identified structures that match the baseline data for *C. chinensis*. The last column indicates the ranks determined by our DP algorithm

### Analysis of time complexity in the DP and iterative DP algorithms

First, we analyzed the time complexity of the DP algorithm described in Fig. 4. Initially, the starting condition of $C(s,w,r)$ executes completely in $O\left( {W_{0}^{{\prime }} R} \right)$ time. The next four for-loops execute in $O\left( {\sum\nolimits_{i = 1}^{n} {W_{0}^{{\prime }} K_{i} R} } \right)$ time, and each iteration of the four for-loops requires $O\left( 1 \right)$ for the side chain information assignments and $O\left( {K_{i} R\log K_{i} R} \right)$ for the max (in Lemma 3) function, which is applied by a quick-sort algorithm. Thus, the time complexity of *DPforCSCCP* is

$$ O\left( {W_{0} \mathop \sum \limits_{i = 1}^{n} \left( {K_{i} R + K_{i} R\log K_{i} R} \right)} \right) = O\left( {W_{0}^{{\prime }} R\left[ {\mathop \sum \limits_{i = 1}^{n} \left( {K_{i} \log K_{i} R} \right)} \right]} \right). $$

In real cases, *R* is a constant. If $K^{{\prime }} = max_{i = 1..n} K_{i}$, the total time complexity can be converted to $O\left( {nW_{0}^{{\prime }} K^{{\prime }} {\text{Rlog}}K^{{\prime }} R} \right)$ by setting $K_{i}$ equal to $K^{{\prime }}$. However, *DPforCSCCP* is not a real polynomial time algorithm. Because each $W_{0}$ is converted into an integer by multiplying by 10^6^ in the DP algorithm, the complexity becomes $O\left( {10^{6} \times nW_{0} K^{{\prime }} R\log K^{{\prime }} R} \right)$. Although $W_{0}^{{\prime }}$ is a single integer number, the actual input $10^{6} W_{0}$ may be exponentially times greater than $nK^{{\prime }}$. Therefore, the main time complexity of *DPforCSCCP* is the cost on the input $10^{6} W_{0}$, and *DPforCSCCP* is a pseudo-polynomial time algorithm.

We then analyzed the time complexity of the *IDPforCSCCP* algorithm. As shown in Fig. 5, the while loop in the program is the same as that in the *DPforCSCCP* algorithm, and it executes completely in $O\left( {nW^{{\prime \prime }} R\bar{K}\log R\bar{K}} \right)$ time, where $\bar{K}$ is the average of $K_{i}$. Herein, we estimate the average value of the target weight of the natural products. In our collected natural products database, a total of 82,847 natural products contain ring structures, and only these compounds were considered in this study. The average total molecular weight of all possible maximal substituents in each compound is $89.42$, where for a given scaffold, the maximal substituent is the side chain with the maximal molecular weight. On average, we can assume that the variable $W^{{\prime \prime }}$ is a constant. Therefore, in an average case, the time complexity in the while loop of *IDPforCSCCP* reduces to $O(89nR\bar{K}\log R\bar{K})$. The rest of the while loop in *IDPforCSCCP* requires only $O(R)$ to check the size and the weight, and thus, we can ignore this step. Finally, we considered the number of executed while loops. For each while loop, *R* is multiplied by 10. In the first while loop, the program executes in $O(89n10R^{{\prime }} \bar{K}\log 10R^{{\prime }} \bar{K})$; in the second while loop, the program executes in $O(89n10^{2} R^{{\prime }} \bar{K}\log 10^{2} R^{{\prime }} \bar{K})$; and so on. Assuming that the while loop is executed *L* times, the total time complexity of *IDPforCSCCP* then becomes $\mathop \sum \nolimits_{i = 1}^{L} \left( {89n10^{i} R\bar{K}\log 10^{i} R\bar{K}} \right)$ for the average case. When *L* is sufficiently small, the *CSCCP* can be ideally solved in polynomial time, on average, using our iterative DP algorithm, *IDPforCSCCP*. We will discuss the value of *L* in the subsequent section. We noted that in the previous *DPforCSCCP* algorithm, even if $W^{{\prime }}$ is set to $89 \times 10^{D}$, the time complexity is still near exponential. Because $W^{{\prime }}$ has to be converted into an integer number and the precision number *D* is typically set to at least 6, the time complexity of the *DPforCSCCP* algorithm can only be reduced to $O\left( {10^{6} \times 89nR\bar{K}\log R\bar{K}} \right)$ for the average case.

The number of while loop iterations (*L*) is the main factor that improves the time performance of the *IDPforCSCCP* algorithm over the *DPforCSCCP* algorithm. We used while loops to overcome the limitation of the number of decimal places of the mass (D) in the *DPforCSCCP* algorithm. According to the analysis of the time complexity in *IDPforCSCCP*, *L* is a dominant factor affecting the time performance of the algorithm. In Table 5, we list the values of *L* executed by the *IDPforCSCCP* algorithm in the four tested natural products. Because the *IDPforCSCCP* algorithm can finish within three loops for all tested cases, three situations in Table 5 are considered: the first situation involves cases in which the *IDPforCSCCP* algorithm finished in only one while loop, the second one involves cases in which the *IDPforCSCCP* algorithm ran to completion in exactly two while loops, and the last represents cases in which the *IDPforCSCCP* algorithm finished in exactly three while loops. The second to fourth columns in Table 5 list the number of executed *CSCCPs* in each tested case based on the three situations. The percentages of the numbers occurring in the three situations are indicated in parentheses. In 99% of the situations, the *IDPforCSCCP* algorithm finished in only one loop. Only a few cases required two or three loops for calculation, and no cases required more than three loops. The *IDPforCSCCP* breaks in the first while loop only when the number of current solutions in which the total weights match to the $W^{{\prime \prime }}$ in the first loop are less than *R*, or when more than *R* optimal solutions was found in the first loop. The main idea behind *IDPforCSCCP* for acceleration of structural elucidation is by removing all decimal digits of molecular weight. In the low incidence of cases where the *IDPforCSCCP* required more than one loop indicates the elimination of decimal digits is unlikely to affect the precision of structure elucidation or the search for optimal solutions. Therefore, the two breaking conditions are satisfied with high incidence in the end of the first while loop.

**Table 5:** Running iterations of the while loop executed using the *IDPforCSCCP* algorithm for our four tested herbs

Tested herbs	# of running iterations executed within one loop	# of running iterations executed in exactly 2 loops	# of running iterations executed in exactly 3 loops
*C. chinensis*	975 (99.9%)	1 (0.1%)	0 (0%)
*O. japonicus*	164 (100%)	0 (0%)	0 (0%)
*P. multiflorum*	1338 (98.5%)	16 (1.2%)	4 (0.3%)
*Angelica* sp.	4084 (100%)	0 (0%)	0 (0%)

The numbers in parentheses are the percentage of occurrence of the three situations listed in the second to fourth columns

Assuming that the number of while loop iterations is only 1, the time complexity of the entire *IDPforCSCCP* algorithm only requires $O\left( {89n\bar{K}R^{{\prime }} \log \bar{K}R^{{\prime }} } \right)$ time for the average case, where $\bar{K} = (\mathop \sum \nolimits_{i = 1}^{n} K_{i} )/n$ and $R^{{\prime }}$ is 100. Thus, the time complexity of *IDPforCSCCP* can be further reduced to $O\left( {8900n\bar{K}\log 100\bar{K}} \right)$ for the average case. Therefore, our novel CASE procedure developed using the iterative DP algorithm can automatically elucidate the unknown structures in complex mixtures within reasonable polynomial time for the average case.

### Time performance comparison with traditional algorithms

In this section, the time performances of the *DPforCSCCP* and *IDPforCSCCP* algorithms were compared with those of traditional methods, including the brute force (BF) method and the branch and bound (BB) strategy. The total execution times of the BF, BB, *DPforCSCCP* and *IDPforCSCCP* algorithms for the elucidation of the main structures in the four natural products are listed in Table 6. The first column represents the tested natural products. The second to fifth columns give the execution time (in seconds) for the BF, BB, *DPforCSCCP*, *and IDPforCSCCP* algorithms, respectively. In *DPforCSCCP*, we set the number of decimal places of the mass ($D$) to 2 because the main structures of the four natural products predicted from *DPforCSCCP* were all matched to the validated structures when the parameter *D* was set to at least 2. More than 3 years of computation time would be required to finish structural elucidation in the *CSCCP* using the BF algorithm for *C. chinensis*, and more than 6 years would be required using the BF algorithm for *P. multiflorum*. The execution times of the BF algorithm for *C. chinensis* and *P. multiflorum* were both extremely large. The main reason for such long execution times was that the BF searching method requires the examination of all combinations of *seed scaffolds* and side chains. The maximal numbers of side chain combinations ($\prod\nolimits_{i = 1}^{n} {K_{i} }$) in *CSCCP* for *C. chinensis* and *P. multiflorum* are 41,249,161,384,704 and 2,429,941,913,502,481. Note that the numbers of *seed scaffolds*, *targeted MWs*, and possible sets of positions on the seed scaffolds were not considered in the maximal numbers. Obviously, the thousands of trillions of calculations in the BF algorithm resulted in an unreasonable execution time for structural elucidation in these two cases. Even if parallel programming is applied to solve the BF algorithm, the method still cannot finish within a reasonable time. The traditional BB method can obviously improve the time performance in the four tested cases. However, the BB algorithm still required more than 1 day for the structural identification of *P. multiflorum* and *Angelica* sp.

**Table 6:** Time performance comparison between the BF, BB, *DPforCSCCP* and *IDPforCSCCP* algorithms

Cases	Execution time seconds (min)			
	BF	BB	*DPfor CSCCP*	*IDPfor CSCCP*
*C. chinensis*	> 3 years	914 (15.2)	3757 (62.6)	17 (0.3)
*O. japonicus*	177 (3.0)	141 (2.4)	12 (0.2)	0.75 (0.01)
*P. multiflorum*	> 6 years	> 1 days	1079 (18.0)	67 (1.1)
*Angelica* sp.	> 80 days	~ 1 days	3919 (65.3)	188 (3.1)

In our *DPforCSCCP* algorithm, the program finished within 1.5 h for all cases. In the case of *P. multiflorum*, which is the most complex example, our DP algorithm reduced the execution from 6 years by BF to 1079 s (18 min). The execution time of the DP algorithm is also much faster than the BB algorithm for all tested cases, except *C. chinensis*. The time performance of the BB algorithm for *C. chinensis* is 4 times faster than that of the *DPforCSCCP* algorithm, whereas the time performance of *DPforCSCC*P algorithm for *O. japonicus*, *P. multiflorum*, and *Angelica* sp. is 12–80 times faster than that of the BB algorithm. For the extreme case of *P. multiflorum*, the execution time of the BB algorithm requires over 1 day, whereas for the *DPforCSCCP* algorithm, only 1079 s (18 min) are needed. This result confirms that our developed *DPforCSCCP* algorithm can be executed in polynomial time for an average case and is faster than the BB algorithm. However, as $D$ increases, the execution time for *DPforCSCCP* may become lower than that for the BB algorithm or even the BF algorithm (Lemma 5). The iterative DP algorithm, *IDPforCSCCP*, can perform the structural elucidation without setting the parameter *D*. To confirm that the *IDPforCSCCP* algorithm outperforms the *DPforCSCCP* algorithm in the four tested cases, we present the execution time of the *IDPforCSCCP* algorithm for the four natural products in Table 6. The execution times range from 12.18 to 3919.70 s (0.2–65.3 min) for *DPforCSCCP* and from 0. 75 to 188.44 s (0.01–3.1 min) for *IDPforCSCCP*. *IDPforCSCCP* finished within 3 min in all cases. On average, the time performance of *IDPforCSCCP* is 67 times better than that of *DPforCSCCP*. For the extreme case of *P. multiflorum*, the *IDPforCSCCP* algorithm reduced the execution time from 6 years by BF to close to 1 min. Our iterative DP algorithm is much more efficient than the BF, BB, and our original DP algorithms.

Several approaches including the BF, BB, *DPforCSCCP* and *IDPforCSCCP* algorithms were applied to solve the CASE problem in this study. The BF and BB algorithms blindly check all combinatorial candidates for the substituted positions in the scaffold, while the two DP algorithms, *DPforCSCCP* and *IDPforCSCCP*, formulate the *CSCCP* in terms of a cost equation and save each solution of each sub-problem for the effective generation of optimal solutions. The *IDPforCSCCP* algorithm can reduce a large number of combinations of side chains to identify the main structures in natural products without the limitation of the number of decimal places of the mass, thus further accelerating the identification procedures. *IDPforCSCCP* would realize the spectroscopist’s dream of fully automated structural elucidation.

### Improvement in the structural elucidation results using the iterative DP algorithm

**Fig. 2:**
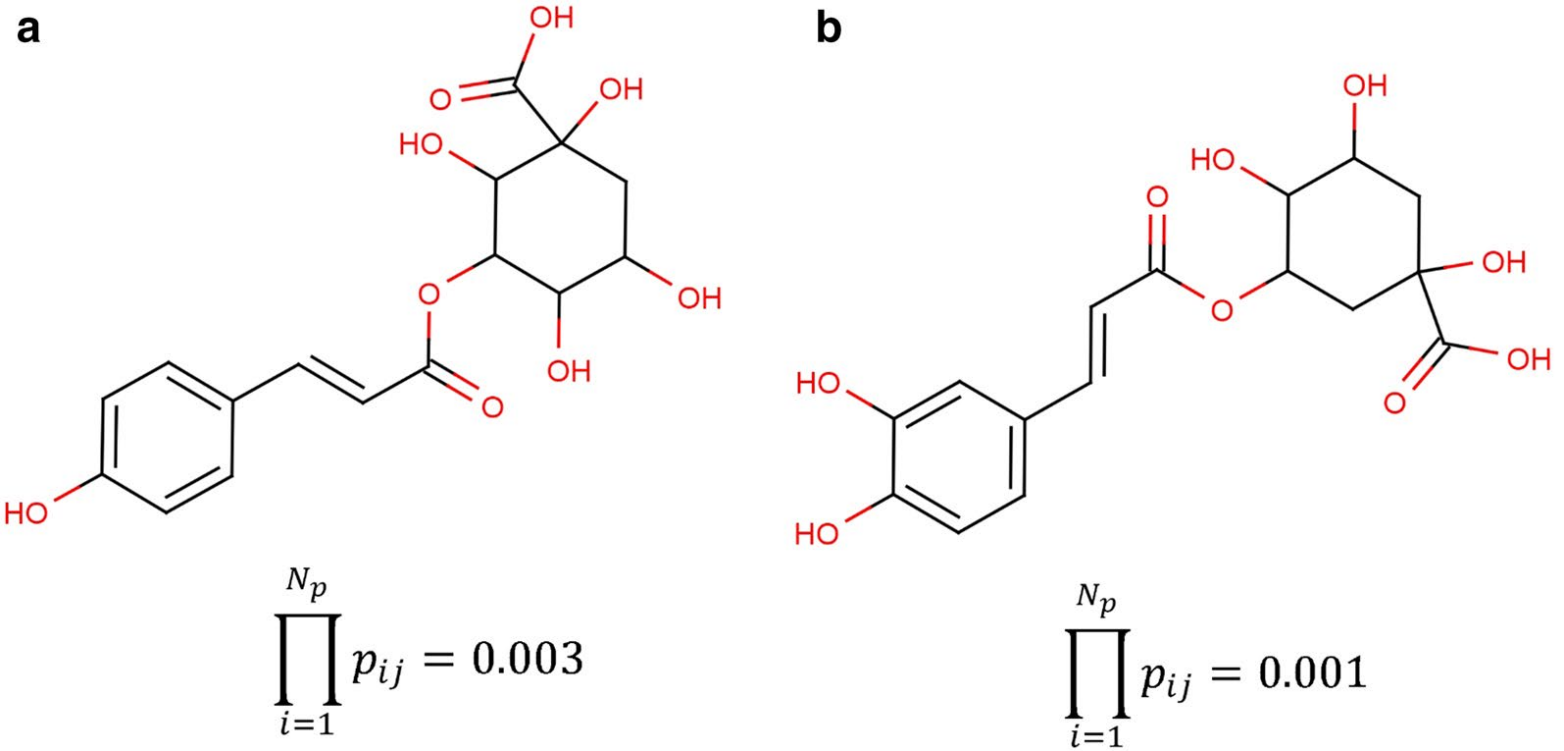
The top-ranking results identified by the branch and bound method with the limitation of *N*_*k*_ = 5 (**a**) and the *IDPforCSCCP* algorithm without limitation of *N*_*k*_ (**b**) for the *targeted MW* of 354.31 in *C. chinensis* with *seed scaffold* 1

Using the iterative DP algorithm, we can search for optimal solutions for structural elucidation without any limitations, compared to the previous study by Harn [29]. A large number of possible sets of positions (*N*_*k*_) that can be linked by the side chains on the *seed scaffolds* could result in an unreasonable execution time for structural elucidation. To reduce the execution time when applying the BB algorithm, the parameter *N*_*k*_ was limited to 5, at most, in Harn’s CASE tool [29]. Thus, the structures identified using Harn’s algorithm may be trapped in a local optimum. In the iterative DP algorithm, the variable *N*_*k*_ does not need to be limited, as our algorithm is much more efficient than the BB algorithm. We took the herb *C. chinensis* as an example to demonstrate the differentiation between our global optimal results and local optimal results determined by the BB algorithm. Table 7 gives the number of structures identified with the BB and *IDPforCSCCP* algorithms for the different *targeted MWs* of *C. chinensis* with the given *seed scaffolds.* The number of structures identified by the BB algorithm ranged from 3 to 18 for scaffold 1 and from 149 to 300 for scaffold 2, while the number identified by *IDPforCSCCP* ranged from 4 to 116 for scaffold 1 and from 1802 to 2006 for scaffold 2. Eight times more structures were recognized by the *IDPforCSCCP* algorithm than by the BB method in this example because *IDPforCSCCP* can maximize the chemical searching space in the *CSCCP*. We also show the top-ranking structures identified by Harn’s method with the limitation of *N*_*k*_ = 5 in Fig. 2a and that identified by the *IDPforCSCCP* algorithm without limitation of *N*_*k*_ in Fig. 2b for the *targeted MW* of 354.31 in *C. chinensis* with *seed scaffold* 1. The estimated probabilities of the potential main structures in *C. chinensis* are also shown below each structure. The *IDPforCSCCP* algorithm can identify a top-ranked structure with higher probability than the BB method. When the value of *N*_*k*_ was limited, the program cannot search all side chain combinations to recognize the optimal structure for a given *targeted MW*. High-performance computing by *IDPforCSCCP* enables complete structure searches without limitation of *N*_*k*_ for identification of the correct main structures in natural products.

**Table 7:** Number of structures identified by the branch and bound and *IDPforCSCCP* algorithm for the four different *targeted MWs* of *C. chinensis* with the given *seed scaffold*s

*Targeted MWs*	Number of identified structures (Brach and bound (*N*_*k*_ = 5), *IDPforCSCCP)*	
	Scaffold 1	Scaffold 2
286.24	(3, 4)	(149, 1936)
302.24	(12, 24)	(196, 1802)
354.31	(3, 29)	(300, 2006)
478.41	(18, 116)	(300, 1995)

Each cell contains two numbers. The first is the number of structures identified by the branch and bound method (*N*_*k*_ = 5), and the second is for the number of structures identified by the *IDPforCSCCP* algorithm without limitation of *N*_*k*_

To demonstrate the actual improvement in the structural elucidation results using the iterative DP algorithm, we compared the identification results of the structural elucidation of *Angelica* sp. Harn’s CASE tool cannot correctly predict twelve structures out of a total of forty-five main compounds in this case. In fact, the overall prediction rate for our *IDPforCSCCP* algorithm increased to 82%. Four additional structures, including Imperatorin, Isoimperatorin, Umbelliprenin, and Ostruthol, were further correctly identified in our study (Additional file 1: Table S3). *IDPforCSCCP* indeed improved both the time performance and prediction accuracy for the structural elucidation of natural products. In fact, *IDPforCSCCP* still failed to predict some main structures since our system can only utilize the collected side chains to construct possible structures on the scaffolds. If additional common or structurally related side chains were manually input to our database, *IDPforCSCCP* would be able to correctly predict these failed structures.

## Conclusions

For the structural elucidation of complex natural products, we defined a new *CSCCP* problem based only on the information obtained from MS spectra. Theoretically, to solve the *CSCCP*, exponential time should be required in the worst-case scenario. We designed a novel CASE algorithm by applying a classical DP algorithm to search for the optimal solutions, and the time complexity was in pseudo-polynomial time. However, the higher precision of the molecular weight required, the higher the time complexity of our DP algorithm, even reaching exponential time. We thus developed an iterative DP algorithm that can be executed in polynomial time for the average case. Four real natural products, *C. chinensis*, *O. japonicus*, *P. multiflorum*, and *Angelica* sp., were applied in our study to verify the results and time performance of our algorithm. The execution time was compared with that of the BF and BB algorithms. Our iterative DP algorithm outperformed the BF and BB programs. In addition, we could really elucidate the correct structures in herbs derived from a previous study. Because the time performance of our algorithm is more efficient than those of the other algorithms, we could search for the real optimal solutions in an acceptable time in the four tested cases without the limitation described in previous studies of the number of combinatorial candidates of the substituted positions in each scaffold. The proposed efficient algorithm provides a new tool for spectroscopists to aid in the structural elucidation of unknown complex mixtures when only MS spectral information is known. A web service built for structural elucidation is freely accessible (http://csccp.cmdm.tw/).

## Methods

### Data Set

Four types of herbs were used to validate the accuracy and time efficiency of our prediction method: *Cuscuta chinensis* (*C. chinensis*), *Ophiopogon japonicus* (*O. japonicus*), *Polygonum multiflorum* (*P. multiflorum*), and *Angelica sp*. A list of possible scaffolds (*seed scaffolds*) for the tested herbs obtained from the preliminary LC–MS identification procedures from the Natural Product Laboratory of the Taiwan Medical and Pharmaceutical Industry Technology and Development Center (PITDC) is shown in Fig. 1. A preliminary identification of the high-intensity mass peaks was also performed, and the possible molecular weights (*targeted MWs*) of the tested herbs derived from the peak tables are listed in Table 1. The possible *seed scaffolds* and the list of *targeted MWs* were used as input in our new CASE program. The procedure is used to elucidate the possible main chemical structures in herbs containing the list of possible *seed scaffolds* to match the peaks corresponding to the given *targeted MWs*. To combine different side chains on the *seed scaffolds* for identification of most of the possible main structures in the herbs, a database containing a list of possible side chains that can attach on the *seed scaffolds* from natural products was generated. The collected natural product database included the Dictionary of Natural Products (DNP) [33], “ZINC natural products” subset of ZINC [34], and the Traditional Chinese Medicine Database (TCMD, updated on 2010-07-14) [35]. The number of natural products that contain ring structures is 82,847, and only these compounds were considered. Furthermore, the Natural Product Laboratory of PITDC has identified a list of main structures of each herb with their LC–MS/MS procedures. The verified main structures of each herb were provided in the results and discussion section and were used as a baseline for the evaluation of our prediction results.

### Definitions of the structural elucidation problem

Our new CASE system identified main structures that matched the *targeted MWs* of herbs by combining the known *seed scaffolds* with a list of possible side chains. The procedure was defined as a Chemical Substituents-Core Combinatorial Problem (*CSCCP*) in the studies. The scaffold (core) of the compounds represented a common substructure of molecules that may have similar biological activities, and a side chain (substituent) denoted a chemical group that is attached to the scaffold. The position of an atom between the scaffold and a side chain is called a substituted position. A compound may have many different substituted positions, and each substituted position on a scaffold can also be linked by many different attached side chains. For each *seed scaffold*, according to the analysis of the relationship between the scaffolds and side chains from our collected natural product database, we can compute the attaching probabilities of the side chains that can link to each substituted positions of that scaffold. The main procedure of our new CASE method utilized this information to elucidate chemical structures, and the formal definition of the *CSCCP* (Definition 1) was as follows.

#### **Definition 1**

Given a chemical scaffold with n possible substituted positions, let $s \in \{ 1,2, \ldots ,n\}$, where $s$ is a variable representing only the considered substituted positions from $1,2, \ldots , s$. *X*_*s*_ is all sets of possible side chains from position $1,2, \ldots , s$. Let $i \in \{ 1,2, \ldots ,s\}$, where *i* represents the *i*th substituted position when the substituted positions from $1,2, \ldots , s$ are considered. $K_{i}$ is the number of side chains that can be linked to the scaffold at position $i$. Therefore, $\left| {X_{s} } \right| = K_{1} K_{2} \ldots K_{s} .$ The $x_{i}$th substituent at position $i$ of the scaffold, where $x_{i} \in \{ 1,2, \ldots ,K_{i} \}$, has a known probability $p_{{i,x_{i} }} \in [0, 1]$, representing its frequency of occurrence in nature, and a molecular weight $m_{{i,x_{i} }}$, which is a nonzero positive floating point number. The problem is the generation of chemical structures that extend a set of possible substituents to the scaffold while satisfying [1] the condition that the product of the probabilities of all extending substituents is maximized and [2] the total molecular weight of the extending substituents is equal to a specified target molecular weight, $W_{0}$. Furthermore, the top *R* optimal solutions of this optimization problem were the output predicted structures of this problem. When *R* is 1, the optimal solution only includes the highest $\prod\nolimits_{i = 1}^{n} {p_{{i,x_{i} }} }$ value in which the total weight is equal to $W_{0}$. R is an integer parameter in the system. Each substituted position on the scaffold can only be extended by one side chain. The CSCCP can be defined as the following floating point linear programming formulation:

1 $$ \mathop {\hbox{max} }\limits_{top R} \mathop \prod \limits_{i = 1}^{n} p_{{i,x_{i} }} $$

2 $$ \mathop \sum \limits_{i = 1}^{n} m_{{i,x_{i} }} = W_{0} $$

$$ x_{i} \in \left\{ {1,2, \ldots ,K_{i} } \right\},\forall i \in \left\{ {1,2, \ldots ,n} \right\} $$

**Fig. 3:**
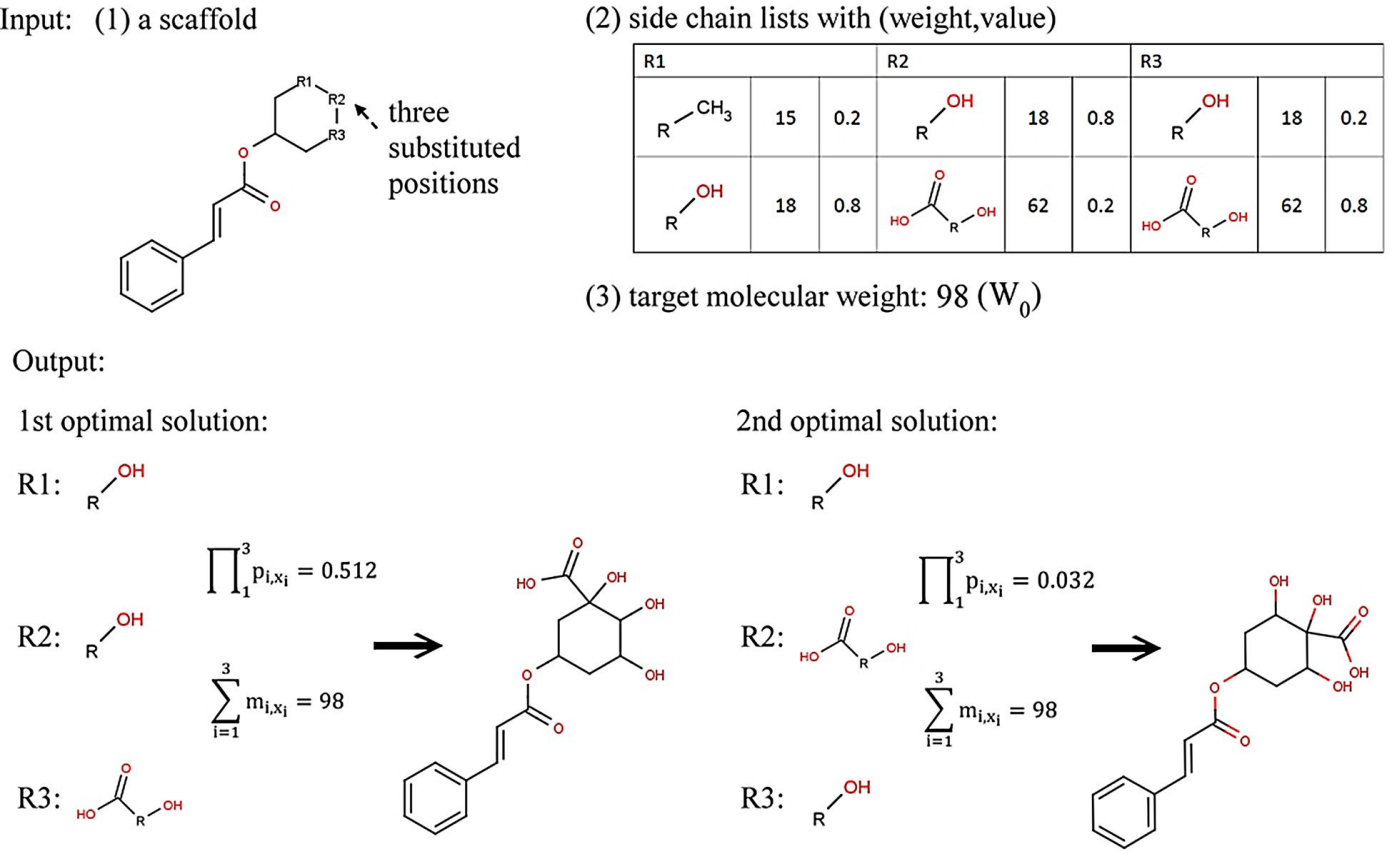
An example structural elucidation defined in our *CSCCP* problem

Figure 3 provides an example illustrating the *CSCCP* optimization problem. Here, given a *seed scaffold* with three substituted positions (*n* = 3), the side chain list for each substituted position (*K*_1_ = 2, *K*_*2*_ = 2, *K*_3_ = 2), and a target molecular weight ($W_{0}$ = 98), each side chain has a pair of molecular weight and occurrence probability values (*m*_1,1_ = 15, *m*_1,2_ = 17, *m*_2,1_ = 17, *m*_2,2_ = 62, *m*_3,1_ = 17, *m*_3,2_ = 62; *p*_1,1_ = 0.2, *p*_1,2_ = 0.8, *p*_2,1_ = 0.8, *p*_2,2_ = 0.2, *p*_3,1_ = 0.2, *p*_3,2_ = 0.8). Two out of a total of eight combinations of side chains generates the structures with the total molecular weight of 98 in this example. The first optimal solution has the highest $\prod\nolimits_{i = 1}^{n} {p_{{i,x_{i} }} }$ value of 0.512, and the second optimal solution has the second highest $\prod\nolimits_{i = 1}^{n} {p_{{i,x_{i} }} }$ value of 0.032. When R is 1, the first optimal solution is the result of predicted structure. In fact, to identify the correct main structures in the natural product, more than one possible *seed scaffold* should be given. Moreover, for a given *seed scaffold*, all combinations of side chains derived from sets of different substituted positions should be considered to generate possible compound candidates. For example, a scaffold has a set of possible substituted positions (for example, in Fig. 1) for R1, R2, and R3 and may have different candidates for other positions. Therefore, the complexity of these conditions makes the *CSCCP* difficult to be completed in a reasonable time.

### DP algorithm

In this study, we first proposed a DP algorithm as our new CASE strategy that can be executed in pseudo-polynomial time complexity to find the optimal solutions in the *CSCCP*. The optimization problem of *CSCCP* has been defined in Definition 1 by Eqs. 1 1 and 2. The *CSCCP* can also be represented by a cost function defined as follows:

#### **Definition 2**

(*CSCCP cost function, C*) In the CSCCP, $n$ denotes the number of substituted positions on a given seed scaffold, and $W_{0}$ is the targeted MW, as defined in Definition 1. A CSCCP cost function $C:\left\{ {s|1 \le s \le n,s \in N} \right\} \times \left\{ {w|1 \le w \le W_{0} ,w \in N} \right\} \times \{ r|1 \le r \le R,r \in N\} \to \left[ {0, 1} \right]$, is defined such that $C(s,w,r)$ represents the highest $r$th value of $\prod\nolimits_{i = 1}^{s} {p_{{i,x_{i} }} }$ when only *s* out of *n* positions are substituted by side chains and the total weight of the selected side chains is equal to *w* $(\sum\nolimits_{i = 1}^{s} {m_{{i,x_{i} }} } = w)$, where *w* is an integer number. If the original molecular weights $W_{0}$ and $m_{{i,x_{i} }}$ are floating point numbers, they are transformed to integers for the following analysis. Therefore, $C(s,w,r)$ corresponds to a sub-problem of the CSCCP since only *s* substituted positions are considered. The *r* highest values of $\prod\nolimits_{i = 1}^{s} {p_{{i,x_{i} }} } ,x_{i} \in \left\{ {1,2, \ldots ,K_{i} } \right\},\forall i \in \left\{ {1,2, \ldots ,s} \right\}$ are denoted as $C(s,w,1:r)$, where “1:*r*” denotes “from 1 to *r*”. The goal of the CSCCP is to find the *R* highest values $C(n,W_{0} ,1:R)$ of $\prod\nolimits_{i = 1}^{n} {p_{{i,x_{i} }} }$ satisfying $\sum\nolimits_{i = 1}^{n} {m_{{i,x_{i} }} } = W_{0} ,$ where $\hbox{``}1:R\hbox{''}$ denotes “from 1 to *R*”.

Therefore, the problem of finding the optimal solutions in the *CSCCP* can be regarded as solving a mathematical procedure of the cost function $C\left( {n,W_{0} ,1:R} \right)$. To compute the cost function, we first need to set the initial configurations of the cost function. The initial condition of $C(s,w,r)$ obeys the following Lemma 1.

#### **Lemma 1**

*In* $C(0,w,r)$ *, for any values of w and r,* $C(0,w,r)$ *is 0, except for the case of* $C\left( {0,0,1} \right),$ *in which* $C(0,0,1)$ *is equal to 1.*

Next, we utilized the dynamic programming strategy to iteratively compute the cost function based on the initial condition defined in Lemma 1. The main concept of the DP method is based on the principle of optimality: for any initial conditions and decisions, the decisions selected over the remaining period must be optimal for the remaining problem, with the states resulting from the previous decisions acting as the initial condition [30]. Therefore, to solve the entire *CSCCP* problem, $C(n,W_{0} ,1:R)$, we must compute the sequence of the sub-problems, $C(s,w,1:R)$ for $s = 1, 2, \ldots , n$, and $w = 1,2, \ldots ,W_{0}$. We used Lemma 2 to represent the relationship between the sub-problems.

#### **Lemma 2**

*Given the values of* $C\left( {s - 1, 1:w, 1} \right)$ *, we can infer the value of* $C(s, w, 1)$ *using the equation below:*

$$ C\left( {s, w, 1} \right) = \mathop {\hbox{max} }\limits_{{x_{s} \in \{ 1,2, \ldots ,K_{s} \} }} \{ p_{{s,x_{s} }} \times C(s - 1, w - m_{{s,x_{s} }} ,1)\} $$

According to the principle of DP, the optimal solution of $C(s, w, 1)$ can be decided by the optimal solutions in the previous step, $C\left( {s - 1, 1:w, 1} \right).$ In other words, the highest values of $\prod\nolimits_{i = 1}^{s - 1} {p_{{i,x_{i} }} }$ matching the molecular weights from 1 to $w$ were obtained when the positions from 1 to $s - 1$ on the scaffold were linked by the appropriate side chains derived from the computation of the DP algorithm. Thus, we can directly evaluate the optimal solutions of $C\left( {s, w, 1} \right)$ based on the known $C\left( {s - 1, 1:w, 1} \right)$ in Lemma 2. Next, we extend Lemma 2 into Lemma 3 to calculate the *highest R optimal solutions*.

#### **Lemma 3**

*If* $C(s,w,1:R$) *are the highest R optimal solutions, with* $s \in \{ 1,2, \ldots ,n\}$ *and* $w \in \{ 0,1, \ldots ,W_{0} \}$ *, then:* $C\left( {s, w, 1:{\text{R}}} \right) = \mathop {\hbox{max} }\limits_{{top R, x_{s} \in \left\{ {1,2 \ldots ,K_{s} } \right\},r \in \{ 1,2 \ldots ,R\} }} \{ p_{{s,x_{s} }} \times C\left( {s - 1, w - m_{{s,x_{s} }} ,{\text{r}}} \right)\}$, *where K*_*s*_ *is the number of possible side chains at the sth substituted position on the given seed scaffold and* $m_{{s,x_{s} }}$ *and* $p_{{s,x_{s} }}$ *are the molecular weight and probability of the* $x_{s}^{th}$ *side chain that can be linked on the sth substituted position.*

Note that the order of the positions used to iteratively calculate the sub-problem, $C\left( {s, w, 1:{\text{R}}} \right)$, will not affect the results of the optimal solutions according to our proven Lemma 4.

#### **Lemma 4**

*If we select any arbitrary order of s substituted positions to calculate* $C(s,w,1:R)$ *, the solutions of* $C(s,w,1:R)$ *are unchanged.*

Considering Lemmas 1–4, we can reasonably conclude Theorem 1.

#### **Theorem 1**

*The highest R optimal solutions in the CSCCP,* $C(n,W_{0} ,1:R)$ *, can be solved by iteratively finding the optimal solutions of* $C(s,w,1:R)$ *for the position s from 1 to n and the molecular weight w from 0 to* $W_{0}$ *based on the initial condition of Lemma 1.*

**Fig. 4:**
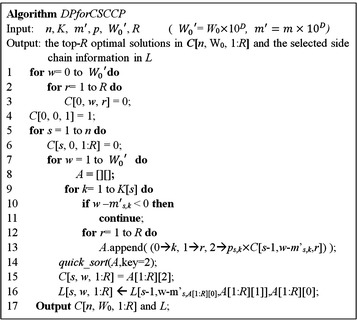
The dynamic programming algorithm for the computation of the Chemical Substituents-Core Combinatorial Problem (CSCCP)

From the above discussions, we designed a new CASE tool to solve the *CSCCP.* The pseudocode of the DP procedure*, DPforCSCCP,* is presented in Fig. 4. According to Lemma 1, lines 1–4 initialize the cost function. Lines 5–16 are the code required to iteratively calculate the cost function of the *CSCCP* for the positions on the *seed scaffold* from 1 to *n* and the molecular weight from 1 to $W_{0}^{'}$ based on Lemmas 2–4, where $W_{0}^{'}$ is the integer format of $W_{0}$. To preserve the precision of the set of optimal solutions determined by the DP algorithm, all molecular weights in ${\text{\{ }}m_{{i,x_{i} }} | i = 1,2, \ldots ,n, x_{i} = 1,2, \ldots ,K_{i} \}$ as well as $W_{0}$ were scaled to integer numbers by multiplying by an appropriate number, *λ*, in order of magnitude. If the number of mass decimal places in *W*_0_ is *D*, we set *λ as* 10^*D*^. The default value of *D* was 6 in our DP algorithm. Then, $W_{0}^{'} = W_{0} *10^{D}$ will retain all the floating point information of the original *W*_0_. Lines 10–11 consider the boundary condition when the molecular weight of the selected side chain is greater than the value of *w*. Lines 9–15 correspond to the calculation of the cost function, $max$, defined in the formula in Lemma 3. The symbol *C* refers to a look-up table of the cost function, which is a 3D matrix in which the index of the weight is in integer format. When followed by brackets, such as in $C[s,w,r]$, $C$ will refer to the look-up table, while when followed by parenthesis, such as in $C(s,w,r)$, $C$ will refer to the previously defined cost function. Another 3D matrix of *L* was used to store the selected side chain information derived from the results of the cost function in line 16. At the end of the DP algorithm, the top *R* highest products of the probabilities from the combinations of the linked side chains were obtained in $C\left[ {n,W_{0} ,1:R} \right]$, where the total molecular weight of the linked side chains was equal to ${\text{W}}_{0}$. The corresponding identified R structures can be generated from the information in matrix *L*.

In the *DPforCSCCP* algorithm, when *D* is too large, the time complexity must be in exponential time. The following lemma shows the lower bound of *D* in this case.

#### **Lemma 5**

*When the number of mass decimal places D is greater than* $\log_{10} \left( {\prod\nolimits_{i = 1}^{n} {K_{i} } } \right)/W_{0}$, *the time complexity of DPforCSCCP is greater than that of the BF algorithm for the CSCCP.*

### Iterative DP algorithm

According to Lemma 5, a larger *D* results in a worse time performance for *DPforCSCCP* that could be even slower than the brute force algorithm. To improve the *DPforCSCCP* algorithm, we introduce in this section a modified algorithm without the limitation of *D*. First, we derived Theorem 2:

#### **Theorem 2**

*Let us assume that all molecular weights* $m_{{i,x_{i} }} \in {\mathbb{R}}$ *are converted into integers* $m_{{i,x_{i} }}^{{\prime \prime }} = m_{{i,x_{i} }} + 0.5 \in {\mathbb{N}}$, $W_{0} \in {\mathbb{R}}$ *is converted to* $W_{0}^{{\prime \prime }} = W_{0} + 0.5 \in {\mathbb{N}}$ *, and* $R$ *is changed to* $R^{{\prime }} > R$ *. Let* $C$ *be the look*-*up table used by DPforCSCCP when the targeted MW is* $W_{0}^{{\prime }} = 10^{D} *W_{0}$ *and* $C^{{\prime }}$ *be the look*-*up table used by IDPforCSCCP when the targeted MW is* $W_{0}^{{\prime \prime }}$ *. Then, if* $R^{{\prime }}$ *is sufficiently large, the set* $C^{{\prime }} \left[ {n, W_{0}^{{\prime \prime }} - 0.5n : W_{0}^{{\prime \prime }} + 0.5(n + 1),R^{{\prime }} } \right]$ *contains all of the values in* $C[n,W_{0}^{{\prime }} ,R]$ *calculated by DPforCSCCP.*

**Fig. 5:**
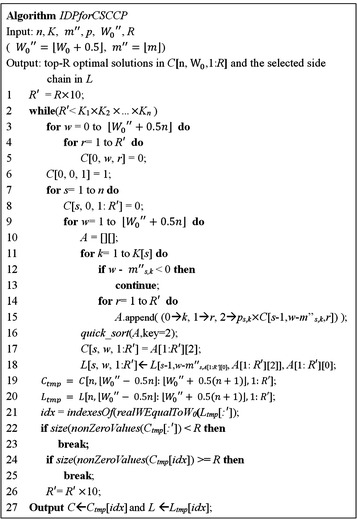
The iterative dynamic programming algorithm for computation of the Chemical Substituents-Core Combinatorial Problem (CSCCP)

Theorem 2 concludes that when a new sufficiently large number $R^{{\prime }}$ is assigned in the DP algorithm, all the optimal solutions of the *CSCCP* still can be evaluated without considering *D*. According to Theorem 2, we proposed the iterative dynamic programming algorithm (*IDPforCSCCP*) depicted in Fig. 5 to efficiently identify the main structures in natural products. Both the *targeted MWs* and molecular weights of the side chains are first directly converted into integer format by truncating all of the floating point information. For example, if $W_{0} = 400.03$, then $W_{0}^{{\prime \prime }} = 400$. Because the main procedure of the DP algorithm for the calculation of the cost function was not modified, the code from line 3 to line 16 in *IDPforCSCCP* is the same as the code in *DPforCSCCP*. However, the code from line 3 to 16 is enclosed by a new while loop to evaluate the cost function based on different ranges of $R^{{\prime }}$. In the while loop starting from line 1 of the *IDPforCSCCP* algorithm, the value of *R’* in the first iteration is set to *R* × 10, and for the following iterations, *R’* is iteratively multiplied by 10 until $R^{{\prime }}$ is greater than $\prod\nolimits_{i = 1}^{n} {K_{i} }$. The code from line 17 to 18 is used to store the values of the cost function and the corresponding side chains. In line 19 and 20, *C*_*tmp*_ and *L*_*tmp*_ are one-dimensional arrays storing all of the elements in $C[n,W_{0}^{{\prime \prime }} - 0.5n:W_{0}^{{\prime \prime }} + 0.5(n + 1),1:R^{{\prime }} ]$ and $L\left[ {n,W_{0}^{{\prime \prime }} - 0.5n:W_{0}^{{\prime \prime }} + 0.5\left( {n + 1} \right),1:R^{{\prime }} } \right],$ respectively. Because we ignore the number of mass decimal places in the *targeted MW* in *IDPforCSCCP*, we use the function *realWEqualToW*_0_ in line 21 to evaluate whether the molecular weights of the identified solutions in floating point format are equal to the original *targeted MW*, $W_{0}$. The composite function *indexesOf(realWEqualToW*_*0*_ *())* in line 21 returns the indexes of the correct optimal solutions in which the real weight is equal to $W_{0}$. When the number of correct optimal solutions in the while loop is greater than *R*, as coded in line 24, the while loop is broken in line 25 because the top *R* optimal solutions have already been identified. In line 24 of Fig. 5, the *nonZeroValues* function takes an array as input and returns the same array without zero values, while the *size* function returns the size of the array. In *IDPforCSCCP*, we should also consider another boundary condition derived from the following Lemma 6.

#### **Lemma 6**

*In the IDPforCSCCP algorithm, if the number of optimal solutions searched in the first iteration is less than R, the set of the searched optimal solutions will not be updated in subsequent iterations.*

According to Lemma 6, we designed the boundary condition in lines 22–23. If the size of the searched optimal solutions is less than *R*, the while loop is also broken. The proofs for Lemmas 1–6 and Theorems 1–2 are all given in the Additional file 1. The source code of the *IDPforCSCCP* algorithm programming in Java was provided in the Additional file 2.

## Additional files

**Additional file 1.** Proof of Lemma 1 to Theorem 6, Tables S1–S4, and a discussion for Table S4. The detailed proofs for Lemma 1, Lemma 2, Lemma 3, Lemma 4, Theorem 1, Lemma 5, Theorem 2, and Lemma 6 are given. **Table S1** The identified structures that match the baseline data for *O. japonicus*. The last column indicates the ranks determined by our DP algorithm. **Table S2** The identified structures that match the baseline data for *P. multiflorum*. The last column indicates the ranks determined by our DP algorithm. **Table S3** The identified structures that match the baseline data for *Angelica* sp. The last column indicates the ranks determined by our DP algorithm. **Table S4** Numbers of searched optimal solutions for *C. chinensis*. Each cell contains two numbers. The first number is that determined when the number of combinatorial candidates for the substituted positions is 5, and the second the value determined in the case without limitation.

**Additional file 2.** The source code of the *IDPforCSCCP* algorithm. The java source code of the *IDPforCSCCP* algorithm was provided in the file.
