# Supplementary material for: An efficient computer-aided structural elucidation strategy for mixtures using an iterative dynamic programming algorithm
Source: J Cheminform. 2017 Nov 15;9:57. doi: 10.1186/s13321-017-0244-9 (PMC5688056; doi:10.1186/s13321-017-0244-9)
Supplement: Supplementary file 1 — Additional file 1. Proof of Lemma 1 to Theorem 6, Tables S1–S4, and a discussion for Table S4. The detailed proofs for Lemma 1, Lemma 2, Lemma 3, Lemma 4, Theorem 1, Lemma 5, Theorem 2, and Lemma 6 are given. Table S1 The identified structures that match the baseline data for O. japonicus. The last column indicates the ranks determined by our DP algorithm. Table S2 The identified structures that match the baseline data for P. multiflorum. The last column indicates the ranks determined by our DP algorithm. Table S3 The identified structures that match the baseline data for Angelica sp. The last column indicates the ranks determined by our DP algorithm. Table S4 Numbers of searched optimal solutions for C. chinensis. Each cell contains two numbers. The first number is that determined when the number of combinatorial candidates for the substituted positions is 5, and the second the value determined in the case without limitation. [file 13321_2017_244_MOESM1_ESM.docx]

Supplementary

**LEMMA 1.**

$\left\{ \begin{aligned} C\left( 0,0,1 \right)=1 \\ C\left( 0,w,r \right)=0 , \forall\left( w,r \right)\neq\left( 0,1 \right) \end{aligned} \right.$

*In* $C(0,w,r)$*, for any values of* $w$ *and* $r$*,* $C(0,w,r)$ *is 0 except for the case of* $C(0,0,1)$*.* $C(0,0,1)$ *is equal to 1.*

PROOF. When none of the side chains are substituted on a scaffold, the total weight of the side chains must be zero. Therefore, the probability of the cost function for $C(0,0,1)$ is 100%. Because the solution for $C(0,0,1)$ is empty, we can conclude that:

$$C\left( 0,0,r \right)= 0, for r = 2,3\ldots,R$$

In addition, when no substituted position is considered, the total weight must be zero. Therefore, the initial cases having nonzero total weights cannot exist, and $C(0,w,r) = 0$*, for* $w \neq0$*, and* $r = 1,2,\ldots R$. From the above equations, the following condition is true: $C(0,w,r) = 0$*,* $r = 1,2,\ldots R$, except for $C(0,0,1) = 1$.

**LEMMA 2.** *As defined,* $C\left( s-1,1:w,1 \right)\mathbb{\in R}$ is *the optimal solution of CSCCP with* $n = s - 1$*,* $W_{0} = w$*. Then the optimal solution* $C(s,w,1)$ *to CSCCP with*$n = s$*,* $W_{0} = w$*, is given by the following equation:*

$C\left( s,w,1 \right)=max\left\{ p_{s,x_{s}}\times C\left( s-1, w-m_{s{,x}_{s}},1 \right) | x_{s}=1,\ldots{,K}_{s} \right\}$*,*

*where K_s_ is the number of side chains in the s^th^ substituted position on the given scaffold, and* $m_{s{, x}_{s}}$ *and* $p_{s,x_{s}}$ *are the molecular weight and probability of x_s_^th^ side chain that can be extended in the s^th^ substituted position, respectively.*

PROOF. Let$N=K_{1}\times K_{2}\times\ldots\times K_{s}$, and $E=\left\{ p_{s{,x}_{s}}\times C\left( s-1, w-m_{s{,x}_{s}},r \right) | r=1\ldots N , x_{s}=1,\ldots{,K}_{s} \right\}$. By definition, $C(s,w,1)$ is the highest value of $\prod_{i=1}^{s} p_{i{,x}_{i}}$ when s side chains are substituted, and the target weight is equal to *w*. Therefore we have:

$$C\left( s,w,1 \right)=\max E=max \left\{ p_{s{,x}_{s}}\times C\left( s-1, w-m_{s{,x}_{s}},r \right) | r=1,\ldots,N , x_{s}=1,\ldots{,K}_{s} \right\}$$

Let $E^{'}=\left\{ p_{s,x_{s}}\times C\left( s-1, w-m_{s{,x}_{s}},1 \right) | x_{s}=1,\ldots{,K}_{s} \right\}$. Trivially we have $E^{'}\subset E$ and $\max E'\in E$.

According to the cost function definition we have:

$$\forall\left( p,m \right)\in\left[ 0, 1 \right]\times\left[ 0, w \right], p\times C\left( s-1, w-m,1 \right)\geq\ldots\geq p\times C\left( s-1, w-m,N \right) (*)$$

Let $y\in E$, then $\exists\left( r,x_{s} \right), y=p_{s{,x}_{s}}\times C\left( s-1, w-m_{s{,x}_{s}},r \right)$. By replacing $p$ by $p_{s{,x}_{s}}$ and $m$ by $m_{s{,x}_{s}}$ in (*) we have $y\leq p_{s{,x}_{s}}\times C\left( s-1, w-m_{s{,x}_{s}},1 \right)\in E'$. Consequently $y\leq\max E'$. So $\max E'$ is the upper bound of *E* and an element of *E*. Therefore $\max E'=\max E$, in other words,

$$C\left( s,w,1 \right)=max\left\{ p_{s,x_{s}}\times C\left( s-1, w-m_{s{,x}_{s}},1 \right) | x_{s}=1,\ldots{,K}_{s} \right\}$$

**LEMMA 3.**

*If* $C(s,w,r)$ *for* $r=1,2,\ldots,R$ *is the highest* $R$ *optimal solutions, where* $1\leq s\leq n$ *and* $0\leq w\leq W_{0}$*, then*

$$C\left( s,w,1:R \right)={max}_{top R}\left\{ p_{s,x_{s}}\times C\left( s-1,w-m_{s,x_{s}}, r \right)| r=1,2,\ldots,R, x_{s}=1,2,\ldots,K_{s}, \right\}$$

*where* $K_{s}$ *is the number of side chains in the s^th^ substituted position on the given seed scaffold, and* $m_{s,x_{s}}$*and* $p_{s,x_{s}}$*are the molecular weight and probability of the x_s_^th^ side chain that can be linked on the s^th^ substituted position.*

PROOF. According to Lemma 2, $C\left( s,w,1 \right)=max\left\{ p_{s,x_{s}}\times C\left( s-1, w-m_{s{,x}_{s}},1 \right) | x_{s}=1,2,\ldots{,K}_{s} \right\}$. Therefore, the values for $\left\{ p_{s,x_{s}}\times C\left( s-1,w-m_{s,x_{s}}, r \right)| x_{s}=1,2,\ldots,K_{s}, r=1,2,\ldots,R \right\}$ could be candidates for the highest *R* probabilities. Next, we will prove that the values in $C$ where $r>R$ do not need to be considered to calculate the highest R probabilities in $C(s,w,1:R)$. According to the definition of the *CSCCP* cost function, the values in $\left\{ C\left( s-1,w-m_{s,x_{s}}, r \right)| , r=1,2,\ldots,R \right\}$ are greater than $C\left( s-1,w-m_{s,x_{s}}, R+1 \right)$ for the *x_s_^th^* side chain. Then, the values in $\left\{ p_{s,x_{s}}\times C\left( s-1,w-m_{s,x_{s}}, r \right)| , r=1,2,\ldots,R \right\}$ are greater than $p_{s,x_{s}}\times C\left( s-1,w-m_{s,x_{s}}, R+1 \right)$ for the *x_s_^th^* side chain. When we only retain the highest *R* optimal solutions in $C\left( s,w,r \right)$, $p_{s,x_{s}}\times C\left( s-1,w-m_{s,x_{s}}, R+1 \right)$ cannot be the top *R* highest probabilities among the set, $\left\{ p_{s,x_{s}}\times C\left( s-1,w-m_{s,x_{s}}, r \right)| x_{s}=1\ldots K_{s}, r=1\ldots R \right\}$. We do not need to consider the values of $r>R$. Therefore, the highest R probabilities among the set $\left\{ p_{s,x_{s}}\times C\left( s-1,w-m_{s,x_{s}}, r \right)| x_{s}=1,2,\ldots{,K}_{s}, r=1,2,\ldots,R \right\}$ are $C(s,w,1:R).$

**LEMMA 4.** *If we select any arbitrary order of s substituted positions to calculate* $C(s,w,1:R)$*, the solutions in* $C(s,w,1:R)$ *are unchanged.*

PROOF. Because the number of all combinations of side chains in $s-1$substituted positions is $K_{1}\times K_{2}\times\ldots\times K_{s-1}$, the upper bound of $R$, $C(s-1,w, K_{1}\times K_{2}\times\ldots\times K_{s-1})$*,* calculates all of the probabilities of all combinations of side chains in $s-1$ substituted positions. In this case, $C(s,w,K_{1}\times K_{2}\times\ldots\times K_{s-1})$ will not lose any candidates regardless of the order of $s-1$ substituted positions selected. However, the cost function only retains the highest $R$ probabilities. Therefore, the value of $R$ is the only factor that could affect the results of arbitrary order. According to Lemmas 1 and 3, when we only retain the top highest $R$ solutions, we can iteratively calculate the optimal solutions in $C(s-1,w,1:R)$ from the initial condition. The solutions in $C(s,w,1:R)$ derived from $C(s-1,w,1:R)$ by lemma 3 must be the optimal solutions when considering $s$ substituted positions. Therefore, the order of $s$ substituted positions will not affect the optimal solutions.

**THEOREM 1.**  *The highest R optimal solutions in the CSCCP,* $C\left( n,W_{0},1:R \right),$*can be solved by iteratively finding the optimal solutions on* $C(s,w,1:R)$ *for the position s from 1 to n, and the molecular weight w from 0 to* $W_{0}$ *based on the initial condition of Lemma 1.*

PROOF. According to Lemmas 1, 2, 3, and 4, we can iteratively calculate the cost function *C* with an arbitrary order of substituted positions until *s* is equal to *n* and *w* is equal to *W_0_*. In the final step, $C(n,W_{0},1:R)$ are simply the highest *R* optimal solutions in the *CSCCP*.

**LEMMA 5.** *When the number of mass decimal digits D is greater than* $\log_{10} \left( \prod_{i=1}^{n} K_{i} \right)/W_{0}$*, the time complexity of DPforCSCCP is greater than that of the brute force algorithm for the CSCCP.*

PROOF. The brute force strategy for *CSCCP* goes through all of the combinations from each of the side chain lists. The time complexity of the brute force algorithm is then $O\left( \prod_{i=1}^{n} K_{i} \right)$. Because the majority of the variables in the time complexity of the *DPforCSCCP* algorithm are *W_0_*, we can only consider the variable *W_0_*. If the time complexity of *DPforCSCCP* is greater than that of the brute force algorithm for the *CSCCP* problem, then:

${{10}^{D}\times W}_{0}>\prod_{i=1}^{n} K_{i}$

Therefore, the inequality can be reduced to:

${{10}^{D}\times W}_{0}>\prod_{i=1}^{n} K_{i}$

${\Rightarrow10}^{D}>\prod_{i=1}^{n} K_{i}/W_{0}$

$\Rightarrow D>{log}_{10}(\prod_{i=1}^{n} K_{i})/W_{0}$

Thus, Lemma 5 is proven.

**THEOREM 2**. *Let us assume that all molecular weights* $m_{i,x_{i}}\mathbb{\in R}$ *are converted into integers*${m^{,,}}_{i,x_{i}}=\left\lfloor m_{i,x_{i}}+0.5 \right\rfloor\mathbb{\in N}$*,* $W_{0}\mathbb{\in R}$ *is converted to* $W_{0}^{''}=\left\lfloor W_{0}+0.5 \right\rfloor\mathbb{\in N}$*, and* $R$ *is changed to*$R’ >R$*. Let* $C$ *be the lookup table used by DPforCSCCP when the targeted MW is weight* $W_{0}'={10}^{D}W_{0},$ *and* $C'$ *be the one used by IDPforCSCCP when the target weight is* $W_{0}''$*. Then if* $R’$*is sufficiently large, the set* $C'\left[ n, \left\lfloor{W_{0}}^{''}-0.5n \right\rfloor: \left\lfloor{W_{0}}^{''}+0.5(n+1) \right\rfloor,R’ \right]$ *contains all of the values in* $C[n,W_{0}',R]$ *calculated by DPforCSCCP.*

PROOF. According to Theorem 1, we can find the top *R’* probabilities values in $C’[n,W_{0}^{''},R’]$. The solutions in $C’[n,W_{0}^{''},R’]$ might not be precisely equal to the solutions in $C[n,W_{0}',R]$ because their corresponding total weight might not equal $W_{0}'$. First, because the upper bound of the difference between $W_{0}^{''} (\sum_{i=1}^{n} {m^{,,}}_{i,x_{i}})$ and $W_{0}$ is $0.5(n+1)$, the optimal solutions could be located between$C'\left[ n, \left\lfloor{W_{0}}^{''}-0.5n \right\rfloor,R’ \right]$ and $C'\left[ n, \left\lfloor{W_{0}}^{''}+0.5(n+1) \right\rfloor,R’ \right]$. Thus, to search the solutions in $C[n,{W_{0}}^{'},R]$*,* the values in $C'\left[ n, \left\lfloor{W_{0}}^{''}-0.5n \right\rfloor: \left\lfloor{W_{0}}^{''}+0.5(n+1) \right\rfloor,R’ \right]$ must all be considered*.* Second, we can test whether the corresponding $\sum_{i=1}^{n} m_{i,x}$ for each $C'\left[ n, \left\lfloor{W_{0}}^{''}-0.5n \right\rfloor: \left\lfloor{W_{0}}^{''}+0.5(n+1) \right\rfloor,R’ \right]$ is equal to $W_{0}$ or not and record the results in the *A* set. If the number of *A* is greater than *R*, the highest *R* probabilities in *A* are $C[n,W_{0},R]$. Third, when *R’* is set to the upper bound in the value of $\prod_{i=1}^{n} K_{i}$, all possible products of the probabilities among the side chains from each substituted positions will be recorded, and $C'\left[ n, \left\lfloor{W_{0}}^{''}-0.5n \right\rfloor: \left\lfloor{W_{0}}^{''}+0.5(n+1) \right\rfloor,R’ \right]$ must contain $C[n,W_{0}^{'},R]$. Therefore, if the size of $R’$is sufficiently large, the set of $C'\left[ n, \left\lfloor{W_{0}}^{''}-0.5n \right\rfloor: \left\lfloor{W_{0}}^{''}+0.5(n+1) \right\rfloor,R’ \right]$ must contain all of the values in $C[n,W_{0}^{'},R]$ calculated by *DPforCSCCP.* Therefore, Theorem 2 is proven.

**LEMMA 6.** *In the IDPforCSCCP algorithm, if the number of searched optimal solutions in the first iteration is less than R, the set of the searched optimal solutions will no more be updated in the subsequent iterations.*

PROOF. Assuming that *R* is set to infinite numbers, the number of searched optimal solutions is fixed in each iteration because *IDPforCSCCP* is a deterministic algorithm. If the number of searched optimal solutions, , in the first iteration is less than $R$*,* the maximum number of possible solutions in the *CSCCP* is . Regardless of the value of $R$, will not be changed. Therefore, ifis less than R in the first iteration, the searched optimal solutions will not be changed in the subsequent iterations.

**Table S1.** The identified structures that match the baseline data for *O. japonicus*. The last column indicates the ranks searched by our DP algorithm.

| weight | Template | Target | rank |
| --- | --- | --- | --- |
| 328.32 |  |  | 5 |
|  |  |  | 1 |
| 342.35 |  |  | 1 |
|  |  |  | 2 |
|  |  |  | 1 |
| 356.33 |  |  | 1 |
| 370.36 |  |  | 3 |

**Table S2.** The identified structures that match the baseline data for *P. multiflorum*. The last column indicates the ranks searched by our DP algorithm.

| Weight | Template | Target | Rank |
| --- | --- | --- | --- |
| 270.24 |  |  | 6 |
| 284.27 |  |  | Non |
| 290.27 |  |  | 1 |
| 432.38 |  |  | 14 |
| 578.53 |  |  | 1 |
| 406.39 |  |  | 1 |
|  |  |  | 4 |

| **Table S3.** The identified structures that match the baseline data for *Angelica sp*. The last column indicates the ranks searched by our DP algorithm. | | |
| --- | --- | --- |
| MW | Scaffold number | Rank order |
| 162.03 | 1 | 1 |
| 186.03 | 2 | 1 |
| 192.04 | 1 | 3 |
| 202.03 | 2 | 1 |
| 216.04 | 2 | 1 |
|  | 2 | 2 |
| 230.09 | 1 | 4 |
| 244.11 | 1 | 7 |
| 246.05 | 2 | 1 |
| 246.09 | 4 | 1 |
| 246.09 | 3 | 1 |
| 270.09 | 2 | 16 |
|  | 2 | 20 |
| 286.08 | 2 | Non |
| 288.10 | 3 | 1 |
| 300.10 | 2 | 34 |
| 316.09 | 2 | Non |
| 328.13 | 3 | 1 |
|  | 4 | 3 |
| 334.11 | 2 | 2 |
| 354.15 | 2 | 83 |
| 360.08 | 2 | Non |
| 360.16 | 1 | Non |
| 366.22 | 1 | 3 |
| 374.14 | 5 | 9 |
| 376.15 | 1 | 24 |
|  | 1 | 38 |
|  | 1 | 38 |
| 378.17 | 1 | 9 |
|  | 1 | 8 |
| 386.14 | 5 | 1 |
|  | 2 | 17 |
|  | 5 | 3 |
| 388.15 | 5 | 8 |
| 402.13 | 5 | Non |
| 414.17 | 5 | 4 |
| 414.20 | 3 | Non |
| 426.17 | 5 | 1 |
| 428.18 | 5 | 6 |
| 518.23 | 2 | Non |
| 546.26 | 2 | 2 |
|  | 2 | 1 |
| 574.29 | 2 | Non |
